# Incidence, prevalence, and risk factors of infectious uveitis and scleritis in the United States: A claims-based analysis

**DOI:** 10.1371/journal.pone.0237995

**Published:** 2020-08-25

**Authors:** Youning Zhang, Sarina Amin, Khristina I. Lung, Seth Seabury, Narsing Rao, Brian C. Toy

**Affiliations:** 1 Department of Ophthalmology, Roski Eye Institute, Keck School of Medicine, University of Southern California, Los Angeles, California, United States of America; 2 Leonard D. Schaeffer Center for Health Policy & Economics, University of Southern California, Los Angeles, California, United States of America; National Taiwan University Hospital, TAIWAN

## Abstract

**Background:**

Ocular inflammation causes significant visual morbidity in the United States, yet little is known about the epidemiology of infectious uveitis and scleritis. This study aims to evaluate the epidemiology of infectious uveitis/scleritis employing a large national medical claims database.

**Methods:**

This was a retrospective, case-control study, employing Optum’s de-identified Clinformatics® Data Mart Database, containing data from 21.5 million privately insured individuals with enrollment for at least 15 months within 2007–2015. Inclusion in the uveitis/scleritis sample required an index uveitis/scleritis diagnosis based on International Classification of Diseases, Ninth Revision (ICD-9) codes. Exclusion criteria included index date within 3 months after intraocular surgery. Rates for uveitis/scleritis were determined by anatomic site. Multivariable logistic regression analyses were performed to determine odds ratios for the incidence and prevalence of uveitis/scleritis by anatomic category.

**Findings:**

Infectious etiologies accounted for less than 20% of uveitis/scleritis, with mean rates of 18.9 (incidence) and 60.6 (prevalence) per 100,000 persons. The mean prevalences of infectious anterior, intermediate, posterior, panuveitis, and scleritis were 27.7, 0.17, 23.4, 4.4, and 4.6, per 100,000, respectively. Overall risk of prevalent infectious uveitis/scleritis increased with age (OR>3.3 for each decade over age 18, p<0.01), female sex (OR = 1.2, p<0.01), non-Hispanic white race (OR<1 for all other races, p<0.01), as well as the East South Central census division (OR = 1.2, p<0.01), comprising Alabama, Kentucky, Missouri, and Tennessee. Medical comorbidities, including HIV infection (OR = 6.4, p<0.01) and rheumatologic disease (OR = 1.9, p<0.01), were common in the infectious uveitis/scleritis cohort.

**Conclusions:**

The incidence and prevalence of infectious uveitis/scleritis in the United States were higher than previously reported estimates but remained lower than in developing countries. Rates varied by age, sex, race, and medical comorbidities, and may reflect differential susceptibility to various infectious agents with disparate geographic distributions within the United States.

## Introduction

Ocular inflammation primarily includes uveitis, affecting the uveal tract, and scleritis, affecting the collagen-rich sclera or its vasculature. Uveitis is estimated to cause 10–15% of blindness in the United States, affecting a wide demographic range of patients [1]. This confers a large socioeconomic impact, costing approximately $242 million annually in the U.S [2]. In spite of this, there are few large studies examining the real-world incidence and prevalence of uveitis and scleritis in the U.S.. In the present study, we employed a large medical claims database to estimate the incidence, prevalence, and risk factors of infectious uveitis and scleritis in the United States.

Infectious etiologies of ocular inflammation are of particular clinical interest, given curative potential if the causative agent is diagnosed and treated in a timely manner. To our knowledge, prior work has not studied the incidence and prevalence of infectious uveitis/scleritis separately from non-infectious ocular inflammation. Several studies have estimated the incidence and prevalence of uveitis overall in the United States, though many of these studies have been limited in scope, focusing exclusively on elderly populations from Medicare or convenience samples from localized geographic areas, unique practice settings, or specialized health system environments [3–9]. As such, the estimated prevalence of any uveitis ranged widely from 58 to 1231 per 100,000 population in the United States. Recent studies of more nationally representative datasets have maintained similar variability, with a recent analysis by Gonzalez et al [10] of 2009–2010 National Health and Nutrition Examination Survey (NHANES) data estimating the prevalence of uveitis to be 540 per 100,000, and a study by Thorne et al [1] of 2012 health insurance claims data demonstrating an overall prevalence of 121 cases per 100,000 adults.

None of these studies focused specifically on infectious ocular inflammation or its risk factors. As a result, very little is known about the epidemiology of infectious uveitis/scleritis in the United States. Somewhat more has been elucidated from studies in the developing world, with known associations including immunocompromised status, high risk sexual behaviors, systemic infection, and geographic location, though it is unclear to what extent these findings generalize to the United States [11, 12]. To fill some of these knowledge gaps, the current study employed a large medical claims database to estimate the incidence, prevalence, and risk factors of infectious uveitis and scleritis in the United States.

## Methods

### Data

Data from Optum’s de-identified Clinformatics® Data Mart Database were reviewed. These de-identified data came from 47 million insured individuals at any point from 2007–2015. For each member, we had access to all medical and pharmacy claims that were filed with their health plan. In addition to basic demographic information (age, race, gender, education level, and income), Optum provided census division (S3 Table) and supplemental socio-economic status (SES) data. The SES data were based on the application of proprietary analytic and demographic models that were created by Optum. Clinical comorbidities were determined employing the methodology described by Elixhauser et al. [13]. The University of Southern California Institutional Review Board determined that this study was exempt from IRB approval. This study complied with the Health Insurance Portability and Accountability Act and adhered to the tenets of the Declaration of Helsinki.

### Selecting overall population and infectious uveitis/scleritis sample

**Fig 1:**
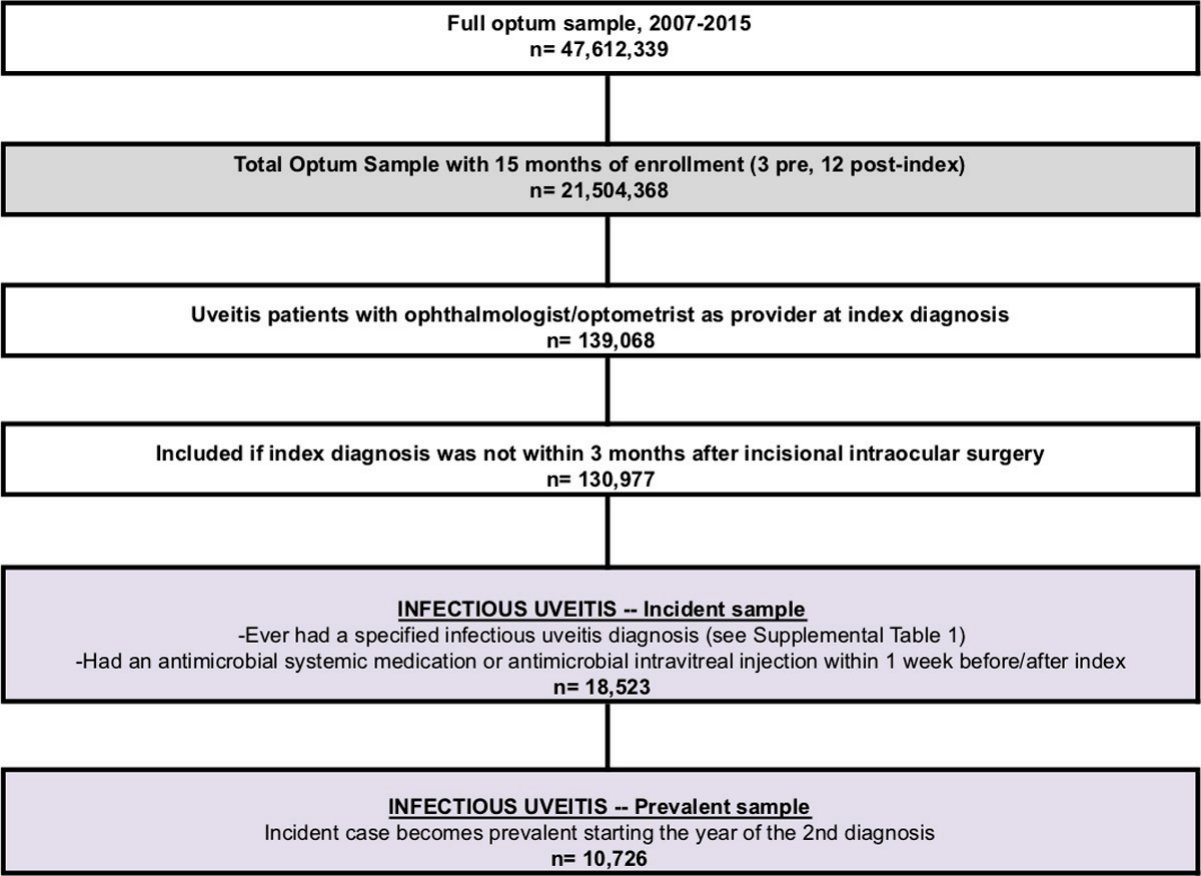
The inclusion flow diagram defining the overall population and the infectious uveitis/scleritis sample.

Fig 1 depicts the inclusion flow diagram defining the overall population and the infectious uveitis/scleritis sample in this study. Inclusion in the overall population required continuous enrollment for at least 15 months within 2007 to 2015 (n = 21,516,133). Inclusion in the uveitis/scleritis sample required an index uveitis/scleritis diagnosis based on International Classification of Diseases, Ninth Revision (ICD-9) codes (S1 Table) by an ophthalmologist or optometrist. We identified the index date for each uveitis/scleritis patient, which was the date of the first claim with a uveitis/scleritis diagnosis. We excluded uveitis/scleritis patients if the index date was within 3 months after incisional intraocular surgery, yielding a final uveitis/scleritis sample size of 130,977 persons. Employing strategies by Borkar et al [14] to improve the validity of our claims data analyses, incident cases of infectious etiology (n = 18,523) were identified by diagnosis with a specifically infectious ICD-9 code or diagnosis with an unspecified uveitis/scleritis ICD-9 code and treatment within 1 week of the index date with intravitreal injection or systemic administration of antimicrobial drug (S2 Table). Cases were considered to be prevalent (n = 10,726) in the year of the second uveitis/scleritis diagnosis. Descriptive statistics for the overall cohort and the infectious uveitis/scleritis sample are reported in Table 1.

**Table 1:** Descriptive statistics for the overall cohort and the infectious uveitis/scleritis sample.

	Total Optum		Any Infectious Uveitis/Scleritis		p-value
***Sample Size*, *n***	21,504,368		18,523		
***Mean age*, *years (sd)***	40.5	(22.7)	57.6	(17.6)	<0.01
***Male*, *n (%)***	10,486,782	(48.77%)	7,580	(40.92%)	<0.01
***Race*, *n (%)***					
Non-Hispanic white	13,601,491	(63.25%)	13,007	(70.22%)	<0.01
Black	1,874,353	(8.72%)	1,712	(9.24%)	0.01
Hispanic	2,267,992	(10.55%)	1,027	(5.54%)	<0.01
Asian	988,291	(4.60%)	397	(2.14%)	<0.01
Unknown or missing	2,772,241	(12.89%)	2,380	(12.85%)	0.86
***Education*, *n (%)***					
Less than high school	133,013	(0.62%)	53	(0.29%)	<0.01
High school only	5,278,876	(24.55%)	4,955	(26.75%)	<0.01
Some college	11,288,872	(52.50%)	9,769	(52.74%)	0.51
4 year college degree or better	4,320,623	(20.09%)	3,418	(18.45%)	<0.01
Unknown or missing	482,984	(2.25%)	328	(1.77%)	<0.01
***Income*, *n (%)***					
$40K-$49k	4,054,808	(18.86%)	4,650	(25.10%)	<0.01
$50K-$99k	5,541,428	(25.77%)	5,590	(30.18%)	<0.01
$100k+	6,660,998	(30.98%)	5,323	(28.74%)	<0.01
Unknown or missing	5,247,134	(24.40%)	2,960	(15.98%)	<0.01
***Census Division*, *n (%)***					
East North Central	3,045,565	(14.16%)	3,741	(20.20%)	<0.01
East South Central	762,789	(3.55%)	1,177	(6.35%)	<0.01
Middle Atlantic	1,449,644	(6.74%)	1,095	(5.91%)	<0.01
Mountain	2,004,201	(9.32%)	1,236	(6.67%)	<0.01
New England	729,613	(3.39%)	648	(3.50%)	0.43
Pacific	2,933,513	(13.64%)	1,207	(6.52%)	<0.01
South Atlantic	5,157,140	(23.98%)	4,832	(26.09%)	<0.01
West North Central	164,916	(0.77%)	30	(0.16%)	<0.01
West South Central	2,186,591	(10.17%)	2,427	(13.10%)	<0.01
Unknown	3,070,396	(14.28%)	2,130	(11.50%)	<0.01
***Smoking*, *n (%)***	312,107	(1.45%)	590	(3.19%)	<0.01
***Comorbidities***					
Congestive Heart Failure	227,486	(1.06%)	550	(2.97%)	<0.01
Cardiac Arrhythmia	503,879	(2.34%)	1,212	(6.54%)	<0.01
Valvular Disease	216,972	(1.01%)	552	(2.98%)	<0.01
Peripheral Vascular Disorders	263,317	(1.22%)	592	(3.20%)	<0.01
Hypertension	2,571,554	(11.96%)	5,484	(29.61%)	<0.01
Other Neurological Disorders	185,502	(0.86%)	381	(2.06%)	<0.01
Chronic Pulmonary Disease	809,582	(3.76%)	1,555	(8.39%)	<0.01
Diabetes	1,230,172	(5.72%)	2,813	(15.19%)	<0.01
Hypothyroidism	697,083	(3.24%)	1,378	(7.44%)	<0.01
Renal Failure	260,077	(1.21%)	682	(3.68%)	<0.01
Liver Disease	133,699	(0.62%)	278	(1.50%)	<0.01
Peptic Ulcer Disease	22,584	(0.11%)	54	(0.29%)	<0.01
AIDS/HIV	21,916	(0.10%)	107	(0.58%)	<0.01
Cancer	360,781	(1.68%)	853	(4.61%)	<0.01
Rheumatologic disease	211,962	(0.99%)	632	(3.41%)	<0.01
Obesity	332,758	(1.55%)	566	(3.06%)	<0.01
Weight Loss	88,064	(0.41%)	235	(1.27%)	<0.01
Fluid and Electrolyte Disorders	212,795	(0.99%)	597	(3.22%)	<0.01
Anemia	193,727	(0.90%)	456	(2.46%)	<0.01
Substance Abuse	107,231	(0.50%)	137	(0.74%)	<0.01
Psychoses	60,462	(0.28%)	118	(0.64%)	<0.01
Depression	763,452	(3.55%)	1,104	(5.96%)	<0.01
Other	152,296	(0.71%)	397	(2.14%)	<0.01

EPO = exclusive provider organization; HMO = health maintenance organization; IND = indemnity; OTH = other; POS = point of service; PPO = preferred provider organization; AIDS = acquired immunodeficiency syndrome; HIV = human immunodeficiency virus

Data for total Optum population is shaded in gray, data for any infectious uveitis/scleritis is shaded in purple.

### Incidence, prevalence, and odds ratios calculations

Incidence and prevalence rates for infectious ocular inflammation between the period of 2007 to 2015 were determined by anatomic site, as well as race, age, and gender. Mean annual incidence and prevalence were calculated employing the methodology of Reeves et al. [6]. Univariable and multivariable logistic regression analyses were performed to determine odds ratios (OR) for the incidence and prevalence of ocular inflammation by anatomic category. Multivariable logistic regression analyses controlled for age, sex, race, income, education, geographic location, insurance product, and medical comorbidities. Adjusted odds ratios were considered significant if they indicated a 25% increase or decrease in relative risk compared with reference and with an associated p-value <0.01. All analyses were conducted on Unix workstations employing SAS 9.4 (SAS Institute; Carey, NC) and Stata 16 (StataCorp; College Station, TX).

## Results

The mean annual rates of any uveitis/scleritis diagnosis within the Optum population having 15 months of continuous enrollment from 2007–2015 were 124.3 (incidence) and 316.4 (prevalence) per 100,000 persons. Infectious etiologies accounted for less than 20% of ocular inflammation, with mean annual rates of 18.9 (incidence) and 60.6 (prevalence) per 100,000 persons. S6 and S7 Tables detail the mean and median 5-year incidence, as well as the annual prevalence during the study period for each of the anatomic categories separately. The infectious uveitis/scleritis cohort differed significantly from the overall Optum population in that the patients were older (57.6±17.6 vs 40.5±22.7 years, p<0.01), with a greater proportion of women (59% vs 51%, p<0.01) and whites (70% vs 63%, p<0.01). Comparing the uveitis/scleritis cohort to the overall Optum sample, the proportion of patients in the East Central, South Atlantic, and West South Central census divisions was higher than the proportion of patients in the Mountain and Pacific census divisions. There was a significantly greater prevalence of comorbid medical conditions in the infectious uveitis cohort, including: smoking (3.2% vs 1.5%, p<0.01), cardiac arrhythmia (6.5% vs 2.3%, p<0.01), hypertension (29.6% vs 12%, p<0.01), chronic pulmonary disease (8.4% vs 3.8%, p<0.01), diabetes (15.2% vs 5.7%, p<0.01), hypothyroidism (7.4% vs 3.2%, p<0.01), renal failure (3.7% vs 1.2%, p<0.01), cancer (4.6% vs 1.7%, p<0.01), rheumatoid arthritis/collagen vascular disease (3.4% vs 1%, p<0.01), and fluid and electrolyte disorders (3.2% vs 1%, p<0.01).

**Fig 2:**
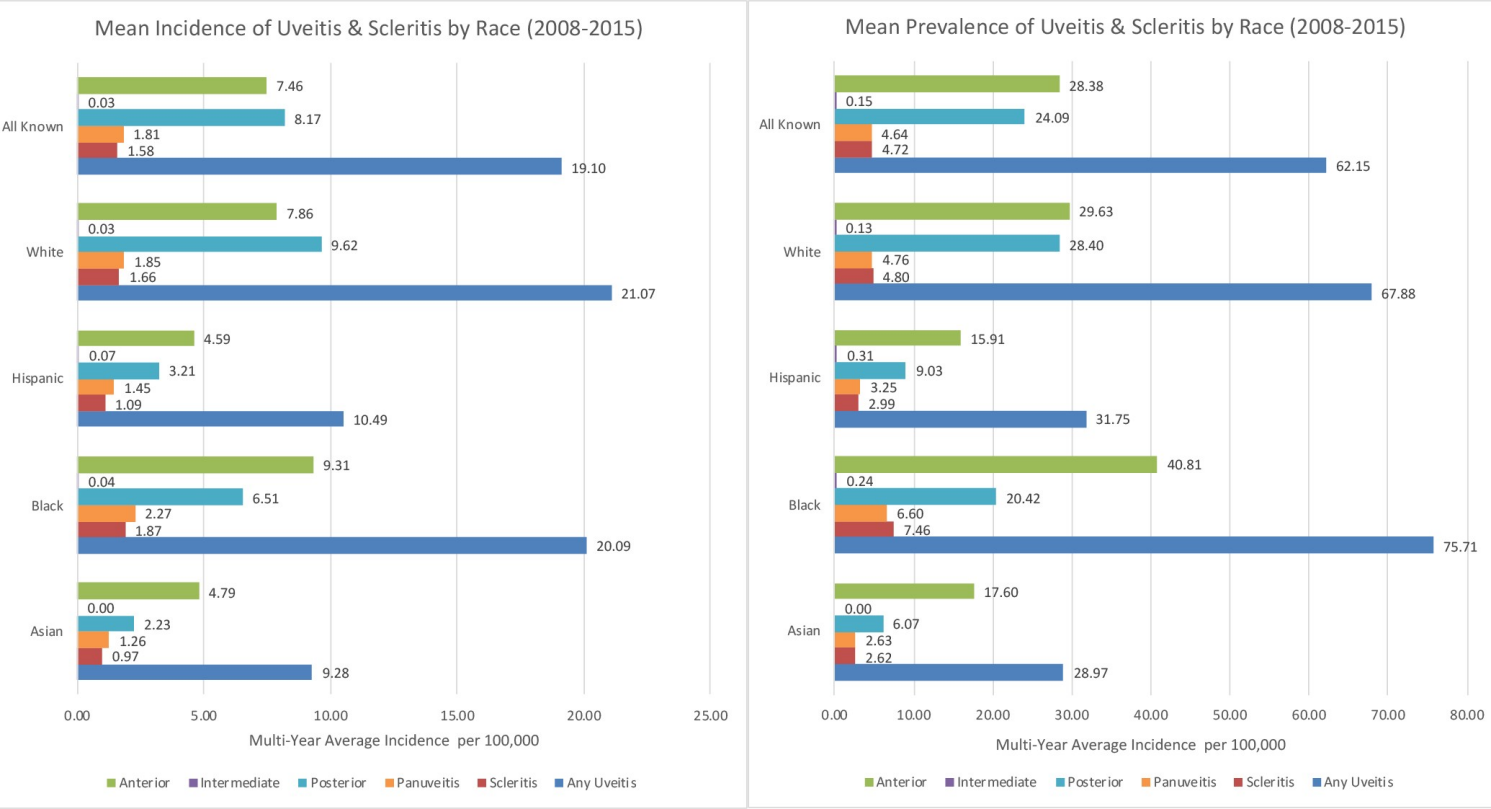
Mean annual incidence and prevalence of uveitis/scleritis by anatomic location and race.

Fig 2 depicts the incidence and prevalence of infectious ocular inflammation by anatomic location and race. Rates of infectious panuveitis and anterior uveitis were highest in African American patients, whereas infectious posterior uveitis was more common in white patients. Infectious intermediate uveitis, while rare overall, had the highest rate in Hispanic patients.

Multivariable logistic regression was performed in parallel for incident and prevalent cases of infectious ocular inflammation overall and by anatomic category. These results are reported below and in Tables 2 and 3.

**Table 2:** Multivariable logistic regression of incident cases of infectious ocular inflammation overall and by anatomic category.

	Any Infectious Uveitis/Scleritis			Scleritis			Anterior Uveitis			Intermediate Uveitis			Posterior Uveitis			Panuveitis		
	n = 18,523			n = 1,540			n = 7,213			n = 33			n = 8,012			n = 1,725		
	%	OR	p-value	%	OR	p-value	%	OR	p-value	%	OR	p-value	%	OR	p-value	%	OR	p-value
***Age categories***																		
0–17	2.6	REF		2.1	REF		3.2	REF		*	REF		1.8	REF		4.3	REF	
18–34	8.4	3.1	<0.01	13.1	5.7	<0.01	9.8	2.9	<0.01	*	6.3	0.09	6.8	3.7	<0.01	5.7	1.2	0.21
35–54	28.5	6.8	<0.01	41.4	11.6	<0.01	28.8	5.6	<0.01	39.4	8.1	0.04	28.3	10.2	<0.01	15.9	2.2	<0.01
55–64	21.3	11.1	<0.01	23.8	14.9	<0.01	19.6	8.3	<0.01	*	12.2	0.02	23.2	18.3	<0.01	17.0	4.8	<0.01
65–74	20.9	13.7	<0.01	11.7	11.0	<0.01	19.3	10.1	<0.01	*	6.0	0.20	23.4	23.7	<0.01	24.2	6.9	<0.01
75+	18.4	16.3	<0.01	7.9	10.1	<0.01	19.2	13.4	<0.01	*	7.2	0.18	16.5	23.3	<0.01	32.9	12.1	<0.01
***Male*, *n (%)***	40.9	0.8	<0.01	31.8	0.5	<0.01	42.2	0.8	<0.01	*	0.4	0.02	41.0	0.8	<0.01	43.7	0.9	0.09
***Race*, *n (%)***																		
Non-Hispanic white	70.2	REF		65.9	REF		66.6	REF		48.5	REF		75.6	REF		64.9	REF	
Black	9.2	0.9	<0.01	10.8	1.2	0.01	11.0	1.2	<0.01	*	2.3	0.17	7.1	0.6	<0.01	10.8	1.0	0.63
Hispanic	5.5	0.7	<0.01	6.6	0.8	0.11	6.3	0.7	<0.01	*	2.7	0.05	4.0	0.6	<0.01	8.3	1.0	0.68
Asian	2.1	0.7	<0.01	2.9	0.8	0.09	2.8	0.8	<0.01	*	1.0	-	1.2	0.5	<0.01	3.0	1.0	0.85
Unknown or missing	12.9	1.1	<0.01	13.7	1.2	0.01	13.4	1.2	<0.01	*	2.7	0.03	12.1	1.1	0.15	13.0	1.1	0.17
***Education*, *n (%)***																		
Less than high school	27.0	REF		20.8	REF		25.2	REF		*	REF		29.9	REF		27.2	REF	
Some college	52.7	1.0	0.13	54.2	1.3	<0.01	53.0	1.1	<0.01	57.6	1.3	0.58	52.2	0.9	<0.01	52.7	1.1	0.06
4 year college degree or better	18.5	1.1	<0.01	23.9	1.5	<0.01	20.0	1.3	<0.01	*	1.3	0.69	16.2	0.9	0.13	17.3	1.3	<0.01
Unknown or missing	1.8	0.8	<0.01	1.1	0.8	0.42	1.8	0.8	0.05	*	1.0	-	1.6	0.7	<0.01	2.8	1.0	0.99
***Income*, *n (%)***																		
$40K-$49k	25.1	REF		18.8	REF		25.0	REF		*	REF		25.1	REF		31.0	REF	
$50K-$99k	30.2	1.1	<0.01	27.7	1.1	0.11	29.5	1.1	0.11	*	0.5	0.22	31.5	1.1	<0.01	29.4	1.0	0.58
$100k+	28.7	1.1	<0.01	37.3	1.3	<0.01	29.4	1.1	0.07	39.4	1.1	0.88	28.0	1.1	<0.01	21.4	0.8	0.03
Unknown or missing	16.0	0.8	<0.01	16.2	0.8	0.04	16.1	0.7	<0.01	*	0.5	0.21	15.4	0.8	<0.01	18.2	0.8	<0.01
***Census Division*, *n (%)***																		
East North Central	20.2	REF		14.6	REF		13.8	REF		*	REF		28.8	REF		12.2	REF	
East South Central	6.4	1.3	<0.01	4.6	1.3	0.07	3.8	1.1	0.21	*	0.8	0.85	9.7	1.4	<0.01	3.4	1.1	0.40
Middle Atlantic	5.9	0.6	<0.01	7.7	1.0	0.71	7.7	1.1	0.15	*	0.8	0.74	3.7	0.3	<0.01	7.1	1.0	0.89
Mountain	6.7	0.5	<0.01	8.1	0.9	0.37	7.7	0.9	0.04	*	2.1	0.20	4.5	0.3	<0.01	11.0	1.3	<0.01
New England	3.5	0.6	<0.01	3.6	1.0	0.75	4.9	1.2	<0.01	*	1.0	-	1.9	0.2	<0.01	5.2	1.2	0.11
Pacific	6.5	0.4	<0.01	7.7	0.7	<0.01	9.1	0.8	<0.01	*	0.9	0.89	3.3	0.1	<0.01	9.5	0.8	0.02
South Atlantic	26.1	0.8	<0.01	28.7	1.1	0.15	28.8	1.2	<0.01	*	0.7	0.51	22.0	0.5	<0.01	32.2	1.5	<0.01
West North Central	13.1	0.2	<0.01	11.2	1.2	0.17	10.6	1.2	<0.01	*	1.0	0.95	16.7	0.9	<0.01	9.1	1.0	0.65
West South Central	11.5	1.0	0.14	13.7	1.0	0.78	13.6	1.2	<0.01	*	0.5	0.33	9.5	0.4	<0.01	10.3	1.0	0.76
***Smoking*, *n (%)***	3.2	1.2	<0.01	2.5	1.0	0.93	3.5	1.3	<0.01	*	1.6	0.65	3.2	1.1	0.06	2.6	0.9	0.37
***Comorbidities***																		
Congestive Heart Failure	3.0	0.9	<0.01	2.2	1.0	0.85	3.4	1.0	0.79	*	1.0	-	2.3	0.8	<0.01	4.8	0.8	0.08
Cardiac Arrhythmia	6.5	1.1	<0.01	4.5	1.2	0.29	7.1	1.2	<0.01	*	1.0	-	5.7	1.0	0.50	10.0	1.1	0.14
Valvular Disease	3.0	1.1	<0.01	2.9	1.5	0.01	2.7	0.9	0.43	*	3.4	0.25	2.9	1.3	<0.01	4.8	1.3	0.04
Peripheral Vascular Disorders	3.2	1.0	0.33	1.8	0.9	0.50	3.3	0.9	0.43	*	2.2	0.47	2.9	0.9	0.45	5.6	1.0	0.73
Hypertension	29.6	1.3	<0.01	24.2	1.4	<0.01	30.1	1.4	<0.01	*	0.9	0.86	28.3	1.2	<0.01	39.1	1.2	<0.01
Other Neurological Disorders	2.1	1.3	<0.01	1.6	1.2	0.32	2.1	1.3	<0.01	*	3.1	0.28	1.8	1.2	0.07	3.4	1.5	<0.01
Chronic Pulmonary Disease	8.4	1.3	<0.01	7.5	1.5	<0.01	9.0	1.4	<0.01	*	1.0	-	8.0	1.3	<0.01	8.6	0.9	0.53
Diabetes	15.2	1.3	<0.01	10.6	1.2	0.09	14.0	1.2	<0.01	*	0.7	0.71	14.6	1.3	<0.01	27.3	2.3	<0.01
Hypothyroidism	7.4	1.1	<0.01	8.4	1.4	<0.01	7.4	1.1	0.05	*	0.5	0.51	7.0	1.1	0.17	9.2	1.1	0.22
Renal Failure	3.7	1.1	0.01	2.1	1.0	0.91	3.9	1.1	0.04	*	5.7	0.05	3.1	1.1	0.34	7.0	1.2	0.07
Liver Disease	1.5	1.2	<0.01	1.7	1.5	0.06	1.6	1.3	0.02	*	6.2	0.02	1.2	1.0	0.77	2.4	1.5	0.01
Peptic Ulcer Disease	0.3	1.1	0.39	*	2.1	0.05	0.2	0.9	0.75	*	1.0	-	0.3	1.2	0.44	0.4	1.1	0.84
AIDS/HIV	0.6	5.6	<0.01	*	2.7	0.03	0.5	4.5	<0.01	*	1.0	-	0.8	8.9	<0.01	0.1	1.1	0.94
Cancer	4.6	1.3	<0.01	3.1	1.1	0.40	4.5	1.2	<0.01	*	4.3	0.02	4.8	1.4	<0.01	5.5	1.1	0.20
Rheumatologic Disease	3.4	1.8	<0.01	4.9	2.8	<0.01	3.9	2.1	<0.01	*	1.9	0.52	2.7	1.4	<0.01	3.5	1.6	<0.01
Obesity	3.1	1.1	<0.01	3.9	1.4	0.03	2.9	1.1	0.40	*	1.4	0.77	3.0	1.1	0.04	3.2	1.0	0.80
Weight Loss	1.3	1.4	<0.01	0.9	1.3	0.40	1.3	1.3	0.03	*	4.6	0.16	1.1	1.3	0.02	2.6	1.9	<0.01
Fluid and Electrolyte Disorders	3.2	1.3	<0.01	2.4	1.1	0.46	3.5	1.4	<0.01	*	3.9	0.11	2.5	1.1	0.23	5.8	1.5	<0.01
Anemia	2.5	1.1	<0.01	2.3	1.2	0.23	2.7	1.2	0.02	*	1.0	-	2.0	1.0	0.59	3.9	1.2	0.17
Substance Abuse	0.7	1.0	0.66	*	0.5	0.11	0.8	1.0	0.96	*	1.0	-	0.6	0.9	0.33	1.3	1.6	0.02
Psychoses	0.6	1.0	0.76	*	1.1	0.89	0.5	0.8	0.29	*	1.0	-	0.7	1.4	0.02	0.7	0.7	0.23
Depression	6.0	1.1	<0.01	8.6	1.6	<0.01	6.2	1.2	<0.01	*	1.2	0.81	5.3	1.0	0.73	5.9	1.1	0.58
Other	2.1	1.2	<0.01	1.6	1.0	0.97	2.1	1.1	0.27	*	1.0	-	1.8	1.1	0.33	4.2	1.6	<0.01

OR = odds ratio; AIDS = acquired immunodeficiency syndrome; HIV = human immunodeficiency virus; * = fewer than 12 individuals; — = cells with values less than 12 individuals were censored

Data for any infectious uveitis/scleritis is shaded in purple. Data for each category of infectious uveitis/scleritis is shaded in green.

Insurance product types were controlled for in the multivariable logistic regression analysis.

**Table 3:** Multivariable logistic regression of prevalent cases of infectious ocular inflammation overall and by anatomic category.

	Any Infectious Uveitis/Scleritis			Scleritis			Anterior Uveitis			Intermediate Uveitis			Posterior Uveitis			Panuveitis		
	n = 10,726			n = 773			n = 4,837			n = 28			n = 4,235			n = 853		
	%	OR	p-value	%	OR	p-value	%	OR	p-value	%	OR	p-value	%	OR	p-value	%	OR	p-value
***Age categories***																		
0–17	2.3	REF		2.1	REF		2.7	REF		*	REF		1.8	REF		1.9	REF	
18–34	7.9	3.3	<0.01	12.0	5.4	<0.01	8.4	2.9	<0.01	*	6.6	0.08	7.0	3.7	<0.01	5.2	2.5	<0.01
35–54	28.9	8.0	<0.01	40.8	11.9	<0.01	28.8	6.5	<0.01	*	5.1	0.12	29.5	10.3	<0.01	15.2	4.9	<0.01
55–64	21.3	12.7	<0.01	24.3	15.8	<0.01	20.1	9.9	<0.01	*	12.5	0.02	23.9	18.2	<0.01	12.2	7.9	<0.01
65–74	21.0	15.6	<0.01	13.1	12.2	<0.01	19.8	11.4	<0.01	*	5.8	0.22	23.2	23.6	<0.01	25.1	15.2	<0.01
75+	18.6	18.4	<0.01	7.8	9.9	<0.01	20.1	15.2	<0.01	*	7.4	0.19	14.6	20.6	<0.01	40.5	31.7	<0.01
***Male*, *n (%)***	40.9	0.8	<0.01	31.7	0.5	<0.01	42.2	0.8	<0.01	*	0.5	0.09	40.6	0.7	<0.01	43.1	0.9	0.30
***Race*, *n (%)***																		
Non-Hispanic white	69.6	REF		64.3	REF		66.3	REF		50.0	REF		75.3	REF		65.8	REF	
Black	9.9	1.0	0.41	12.4	1.4	<0.01	11.7	1.3	<0.01	*	1.9	0.36	7.0	0.6	<0.01	11.6	1.2	0.22
Hispanic	5.5	0.7	<0.01	7.2	0.9	0.66	6.1	0.7	<0.01	*	3.2	0.03	4.0	0.6	<0.01	8.0	0.9	0.66
Asian	2.2	0.7	<0.01	2.5	0.7	0.13	2.8	0.8	0.02	*	1.0	-	1.3	0.5	<0.01	2.6	0.9	0.55
Unknown or missing	12.8	1.2	<0.01	13.6	1.3	0.04	13.2	1.2	<0.01	*	2.5	0.09	12.4	1.1	0.06	12.1	1.0	0.99
***Education*, *n (%)***																		
Less than high school	26.6	REF		23.9	REF		24.6	REF		*	REF		29.1	REF		27.4	REF	
Some college	53.7	1.1	0.01	53.4	1.2	0.14	53.8	1.1	<0.01	60.7	1.0	0.97	53.3	1.0	0.34	54.4	1.1	0.15
4 year college degree or better	18.1	1.1	<0.01	21.4	1.3	0.02	20.0	1.3	<0.01	*	0.6	0.43	16.1	1.0	0.39	14.9	1.2	0.23
Unknown or missing	1.7	0.8	<0.01	1.3	0.9	0.67	1.7	0.8	0.03	*	1.0	-	1.4	0.7	<0.01	3.3	1.2	0.50
***Income*, *n (%)***																		
$40K-$49k	25.4	REF		21.2	REF		25.2	REF		*	REF		24.8	REF		32.6	REF	
$50K-$99k	30.5	1.1	0.01	28.6	1.1	0.52	29.9	1.1	0.10	*	0.7	0.55	31.7	1.1	0.05	30.3	1.0	0.80
$100k+	28.5	1.1	0.01	34.2	1.2	0.16	29.4	1.1	0.07	42.9	1.7	0.35	28.3	1.1	0.04	19.2	0.9	0.29
Unknown or missing	15.6	0.7	<0.01	16.0	0.8	0.03	15.5	0.7	<0.01	*	0.4	0.23	15.2	0.8	<0.01	17.9	0.8	0.02
***Census Division*, *n (%)***																		
East North Central	19.9	REF		15.0	REF		14.0	REF		*	REF		29.2	REF		11.4	REF	
East South Central	5.7	1.2	<0.01	4.8	1.2	0.24	3.3	0.9	0.53	*	1.0	0.97	9.0	1.3	<0.01	3.8	1.3	0.15
Middle Atlantic	5.8	0.6	<0.01	6.1	0.8	0.23	7.6	1.0	0.59	*	1.0	0.98	3.6	0.3	<0.01	6.7	1.0	0.96
Mountain	6.6	0.5	<0.01	6.6	0.7	0.06	7.8	0.9	0.10	*	2.1	0.27	4.3	0.2	<0.01	10.7	1.4	0.03
New England	3.9	0.7	<0.01	3.5	0.9	0.58	5.3	1.3	<0.01	*	1.0	-	1.8	0.2	<0.01	6.2	1.5	0.02
Pacific	6.7	0.4	<0.01	6.7	0.6	<0.01	9.3	0.8	<0.01	*	1.2	0.77	3.0	0.1	<0.01	10.2	0.9	0.53
South Atlantic	26.7	0.8	<0.01	29.9	1.1	0.36	29.3	1.2	<0.01	*	0.9	0.88	22.5	0.5	<0.01	30.6	1.6	<0.01
West North Central	13.4	0.2	<0.01	12.6	1.3	0.09	10.6	1.1	0.04	*	0.9	0.88	17.2	0.9	<0.01	11.0	1.4	0.02
West South Central	11.2	1.0	0.84	14.8	1.1	0.57	12.7	1.1	0.06	*	0.4	0.30	9.4	0.4	<0.01	9.4	1.1	0.55
***Smoking*, *n (%)***	3.2	1.1	0.03	2.5	0.9	0.74	3.5	1.2	<0.01	*	2.2	0.46	3.1	1.1	0.29	2.7	0.9	0.59
***Comorbidities—12 months pre-index***																		
Congestive Heart Failure	3.0	0.9	0.04	1.3	0.6	0.09	3.6	1.0	0.87	*	1.0	-	2.2	0.8	0.02	5.6	0.9	0.51
Cardiac Arrhythmia	6.7	1.1	<0.01	4.1	1.1	0.57	7.6	1.3	<0.01	*	1.0	-	5.5	1.1	0.49	10.3	1.0	0.98
Valvular Disease	3.0	1.1	0.09	2.6	1.4	0.14	2.8	0.9	0.37	*	4.1	0.18	2.8	1.3	<0.01	4.9	1.2	0.28
Peripheral Vascular Disorders	3.2	0.9	0.22	1.9	1.0	0.95	3.4	0.9	0.39	*	2.7	0.38	2.6	0.9	0.30	5.9	1.0	0.84
Hypertension	30.3	1.3	<0.01	23.9	1.3	<0.01	31.2	1.4	<0.01	*	0.9	0.82	28.3	1.3	<0.01	40.7	1.2	0.05
Other Neurological Disorders	1.9	1.1	0.08	2.1	1.6	0.06	2.0	1.1	0.25	*	4.1	0.18	1.5	1.0	0.91	3.4	1.3	0.23
Chronic Pulmonary Disease	8.5	1.3	<0.01	7.4	1.4	0.02	9.5	1.5	<0.01	*	1.0	-	7.4	1.2	<0.01	8.9	0.9	0.42
Diabetes	14.8	1.3	<0.01	10.3	1.1	0.52	14.1	1.1	<0.01	*	1.0	0.96	14.1	1.3	<0.01	26.5	2.0	<0.01
Hypothyroidism	7.6	1.1	<0.01	8.4	1.4	<0.01	7.6	1.1	0.09	*	0.7	0.69	7.1	1.1	0.09	8.9	1.0	0.99
Renal Failure	3.7	1.1	0.04	2.1	1.0	0.94	4.1	1.1	0.08	*	2.8	0.36	2.8	1.0	0.82	7.9	1.2	0.14
Liver Disease	1.7	1.3	<0.01	1.7	1.4	0.21	1.6	1.2	0.06	*	3.5	0.24	1.5	1.3	0.03	2.5	1.5	0.07
Peptic Ulcer Disease	0.3	1.2	0.30	0.4	1.8	0.34	0.2	0.9	0.69	*	1.0	-	0.3	1.5	0.16	*	1.4	0.45
AIDS/HIV	0.7	6.4	<0.01	0.4	3.2	0.05	0.6	4.8	<0.01	*	1.0	-	1.0	11.2	<0.01	*	1.1	0.90
Cancer	4.9	1.3	<0.01	2.8	1.0	0.96	4.9	1.3	<0.01	*	5.6	0.01	4.8	1.4	<0.01	6.7	1.3	0.06
Rheumatoid Arthritis/collagen	3.6	1.9	<0.01	5.0	2.9	<0.01	4.2	2.2	<0.01	*	2.5	0.37	2.5	1.3	<0.01	4.0	1.7	<0.01
Obesity	2.9	1.1	0.41	4.1	1.5	0.03	2.8	1.0	0.78	*	1.0	-	2.9	1.1	0.46	2.9	1.0	0.96
Weight Loss	1.3	1.4	<0.01	1.0	1.5	0.30	1.3	1.2	0.13	*	6.9	0.08	1.1	1.4	0.02	3.0	1.9	<0.01
Fluid and Electrolyte Disorders	3.5	1.4	<0.01	2.3	1.1	0.64	3.9	1.5	<0.01	*	2.3	0.46	2.6	1.2	0.15	7.3	1.9	<0.01
Anemia	2.5	1.1	0.10	2.5	1.4	0.20	2.7	1.2	0.07	*	1.0	-	1.8	0.9	0.56	4.5	1.2	0.24
Substance Abuse	0.7	0.9	0.43	0.8	1.0	0.98	0.8	1.0	0.82	*	1.0	-	0.4	0.6	0.05	1.5	1.8	0.05
Psychoses	0.6	1.0	0.87	0.4	0.7	0.62	0.5	0.8	0.32	*	1.0	-	0.7	1.3	0.13	*	0.8	0.65
Depression	5.9	1.1	0.03	7.6	1.4	0.02	6.2	1.2	0.02	*	0.8	0.78	5.0	0.9	0.34	7.0	1.3	0.09
Other	2.2	1.1	0.06	1.7	1.2	0.58	2.2	1.1	0.63	*	1.0	-	1.8	1.1	0.30	4.5	1.6	0.01

OR = odds ratio; AIDS = acquired immunodeficiency syndrome; HIV = human immunodeficiency virus; * = fewer than 12 individuals; — = cells with values less than 12 individuals were censored

Data for any infectious uveitis/scleritis is shaded in purple. Data for each category of infectious uveitis/scleritis is shaded in green.

Insurance product types were controlled for in the multivariable logistic regression analysis.

Viral and toxoplasmosis infections were among the most commonly identified causative etiologies (Table 4). Notably among viral etiologies, varicella zoster virus was more common than herpes simplex virus. Although toxoplasmosis represented the most common parasitic causative organism when determined by medication code, histoplasmosis was the most commonly identified by diagnosis code.

**Table 4:** Causative organisms of infectious ocular inflammation by diagnosis and medication codes.

	Sample size, n
Specified by ICD-9 Diagnosis Code	
Parasite	
*Histoplasma*	5976
*Toxoplasma*	1104
*Other*	28
Viral	
*VZV*	1129
*HSV*	442
*Other*	577
Unspecified	
*infectious endophthalmitis*	1610
*infectious iridocyclitis*	733
Mycobacterial	36
Syphilis	36
**Specified by NDC or J Medication Code**	
Viral	3854
Bacterial	2943
Toxoplasma	786
Fungal	542
Mycobacterial	49
Parasite (excluding toxoplasma)	33

ICD = International Classification of Diseases; VZV = varicella zoster virus; HSV = herpes simplex virus; NDC = National Drug Code

### Overall

The mean annual incidence and prevalence of any uveitis was 18.9 per 100,000 and 60.6 per 100,000, respectively. The mean 5-year incidence of any uveitis was 85.4 per 100,000, and the prevalence at year 5 of our study (2011) was 49.9 per 100,000. The risk of infectious uveitis incidence and prevalence increased with age. Men were less likely to develop infectious uveitis (OR = 0.8, p<0.01, 95% CI [0.75, 0.80]). Non-Hispanic whites were more likely to develop infectious uveitis compared with all other races (OR <1 for all other races, p<0.01). Education and income level did not have an impact on risk of developing infectious uveitis. Medical co-morbidities, such as smoking (OR = 1.2, p<0.01, 95% CI [1.06, 1.26]), hypertension (OR = 1.3, p<0.01, 95% CI [1.21, 1.31]), neurological disorders (OR = 1.3, p<0.01, 95% CI [1.13, 1.39]), chronic pulmonary disease (OR = 1.3, p<0.01, 95% CI [1.22, 1.36]), diabetes (OR = 1.3, p<0.01, 95% CI [1.28, 1.40]), acquired immunodeficiency syndrome (AIDS)/ Human Immunodeficiency Virus (HIV) infection (OR = 5.6, p<0.01, 95% CI [4.58, 6.73]), cancer (OR = 1.3, p<0.01, 95% CI [1.19, 1.37]), rheumatologic disease (OR = 1.8, p<0.01, 95% CI [1.67, 1.96]), weight loss (OR = 1.4, p<0.01, 95% CI [1.20, 1.56]), and fluid and electrolyte disorders (OR = 1.3, p<0.01, 95% CI [1.16, 1.39]) were associated with higher risk in incidence of infectious uveitis.

### Anterior uveitis

Of the anatomic categories of infectious uveitis, anterior uveitis had the second highest mean annual incidence (7.5 per 100,000) and highest mean annual prevalence (27.7 per 100,000). African Americans had the highest mean annual incidence and prevalence of infectious anterior uveitis (9.3 and 40.8 per 100,000, respectively). Compared with whites, the adjusted odds ratios for the incidence of infectious anterior uveitis among races were as follows: 1.2 for African Americans (p<0.01, 95% CI [1.06, 1.25]), 0.7 for Hispanics (p<0.01, 95% CI [0.67, 0.81]), and 0.8 for Asians (p<0.0, 95% CI [0.68, 0.91]). Men (OR = 0.8, p<0.01, 95% CI [0.79, 0.87]) were less likely to develop infectious anterior uveitis compared with women. The risk of developing infectious anterior uveitis tended to increase with age, education level, and among patients living in New England. Several clinical comorbidities were significantly associated with an increased risk of incident infectious anterior uveitis: smoking (OR = 1.3, p<0.01, 95% CI [1.14, 1.48]), HIV/AIDS (OR = 4.5, p<0.01, 95% CI [3.31, 6.24]), and rheumatologic disease (OR = 2.1, p<0.01, 95% CI [1.88, 2.39]).

### Intermediate uveitis

Infectious intermediate uveitis was rare, with a mean annual incidence and prevalence of 0.03 and 0.17 per 100,000, respectively. Among racial categories, its incidence and prevalence were highest in Hispanics (0.07 and 0.31 per 100,000, respectively), with a trend toward higher risk in incidence (OR = 2.7, p = 0.05, 95% CI [1.02, 7.39]) and prevalence (OR = 3.2, p = 0.03, 95% CI [1.15, 8.75]). Patients aged between 55 and 64 years also had a trend toward increased risk of infectious intermediate uveitis (incidence OR = 12.2, p = 0.02, 95% CI [1.49, 100.73]; prevalence OR = 12.5, p = 0.02, 95% CI [1.51, 103.04]). There was no significant effect of education or income levels on the risk of infectious intermediate uveitis. There was a trend toward increased incidence (OR = 2.1, p = 0.19, 95% CI [0.68, 6.69]) and prevalence (OR = 2.1, p = 0.27, 95% CI [0.57, 7.59]) in patients from the Mountain region, but this did not meet statistical significance. Similarly, the risk of incident infectious intermediate uveitis trended higher in comorbid renal disease (OR = 5.7, p = 0.05, 95% CI [1.04, 31.15]), hepatic disease (OR = 6.2, p = 0.02, 95% CI [1.34, 28.93]), and cancer (OR = 4.3, p = 0.02, 95% CI [1.21, 15.43]). A comorbid cancer diagnosis was also associated with a significantly increased risk of prevalent infectious intermediate uveitis (OR = 5.6, p<0.01, 95% CI [1.54, 20.39]).

### Posterior uveitis

Infectious posterior uveitis had the highest mean annual incidence (8.0 per 100,000) and second highest mean annual prevalence (23.4 per 100,000) among infectious uveitis anatomic categories. Non-Hispanic whites had the highest incidence and prevalence of posterior uveitis (9.6 per 100,000 and 28.4 per 100,000, respectively). This was significantly higher than all other racial groups (OR<1 for all other races in comparison to non-Hispanic whites, p<0.01). The risk of developing infectious posterior uveitis increased with age. Level of education and income had no significant association with infectious posterior uveitis. Geographically, the risk of developing infectious posterior uveitis was highest in the East South Central and East North Central regions, whereas the risk was significantly lower in the Mid Atlantic (incidence OR = 0.3, p<0.01, 95% CI [0.23, 0.30]; prevalence OR = 0.3, p<0.01, 95% CI [0.22, 0.31]), Mountain (incidence OR = 0.3, p<0.01, 95% CI [0.22, 0.28]; prevalence OR = 0.2, p<0.01, 95% CI [0.20, 0.27]), New England (incidence OR = 0.2, p<0.01 95% CI [0.19, 0.26]; prevalence OR = 0.2, p<0.01, 95% CI [0.17, 0.27]), South Atlantic (incidence OR = 0.5, p<0.01, 95% CI [0.44, 0.50]; prevalence OR = 0.5, p<0.01, 95% CI [0.43, 0.52]) and West South Central (incidence OR = 0.4, p<0.01, 95% CI [0.11, 2.07]; prevalence OR = 0.4, p<0.01, 95% CI [0.35, 0.45]) regions. Smoking conferred a slightly higher but not statistically significant risk of infectious posterior uveitis incidence (OR = 1.1, p = 0.06, 95% CI [0.92, 1.32]). Among medical comorbidities, HIV/AIDS conferred the highest risk of incident (OR = 8.9, p<0.01, 95% CI [6.86, 11.43]) and prevalent (OR = 11.2, p<0.01, 95% CI [8.25, 15.30]) infectious posterior uveitis.

### Panuveitis

The mean annual incidence of infectious panuveitis was 1.8 per 100,000, and its mean annual prevalence was 4.4 per 100,000. African Americans had the highest incidence and prevalence of infectious panuveitis (2.3 per 100,000 and 6.6 per 100,000, respectively), though this did not translate into an increased relative risk after multivariable adjustment. The risk of infectious panuveitis increased with age, whereas it was similar across different income and education levels. There was a trend toward increased risk of incident infectious panuveitis in the South Atlantic and Mountain regions (OR = 1.5, p<0.01, 95% CI [1.27, 1.77] and OR = 1.3, p<0.01, 95% CI [1.07, 1.60], respectively), whereas this risk trended to decrease in the Pacific region (OR = 0.8, p = 0.02, 95% CI [0.62, 0.96]). Smoking did not show any increased or decreased risk association with infectious panuveitis (incidence OR = 0.9, p = 0.37, 95% CI [0.64, 1.18]; prevalence OR = 0.9, p = 0.59, 95% CI [0.58, 1.36]). Among medical comorbidities, diabetes (incidence OR = 2.3, p<0.01, 95% CI [2.01, 2.57]; prevalence OR = 2, p<0.01, 95% CI [1.64, 2.32]), rheumatologic disease (incidence OR = 1.6, p<0.01, 95% CI [1.23, 2.07]; OR = 1.7, p<0.01, 95% CI [1.18, 2.40]), weight loss (incidence OR = 1.9, p<0.01, 95% CI [1.37, 2.56]; prevalence OR = 1,9, p<0.01, 95% CI [1.28, 2.91]), and fluid/electrolyte disorders (incidence OR = 1.5, p<0.01, 95% CI [1.23, 1.95]; prevalence OR = 1.9, p<0.01, 95% CI [1.40, 2.52]) were significantly associated with an increased risk of both incident and prevalent infectious panuveitis.

### Scleritis

Infectious scleritis had a mean annual incidence and prevalence of 1.6 and 4.6 per 100,000, respectively, with a similar incidence across races and higher prevalence among African Americans (7.5 per 100,000). African American race also conferred an increased risk of infectious scleritis (incidence OR = 1.2, p = 0.01, 95% CI [1.05, 1.50]; prevalence OR = 1.4, p<0.01, 95% CI [1.08, 1.72]). Scleritis had the highest risk of incidence (OR = 14.9, p<0.01, 95% CI [10.37, 21.31]) and prevalence (OR = 15.8, p<0.01, 95% CI [9.43, 26.44]) in patients between 55 to 64 years of age. There was a trend towards higher risk of incidence and prevalence with higher education and income level. There was a decreased risk of incidence (OR = 0.7, p<0.01, 95% CI [0.56, 0.90]) and prevalence (OR = 0.6, p<0.01, 95% CI [0.44, 0.87]) of infectious scleritis in the Pacific region. Smoking did not confer a higher risk of infectious scleritis after multivariable adjustment. Comorbid rheumatologic disease was associated with a significantly higher risk of incidence (OR = 2.8, p<0.01, 95% CI [2.23, 3.59]) and prevalence (OR = 2.9, p<0.01, 95% CI [2.08, 4.01]) for infectious scleritis.

## Discussion

A large, national medical claims database was analyzed to perform a comprehensive, longitudinal epidemiologic study of infectious uveitis and scleritis in the United States. Our findings provide new data regarding the real-world incidence and prevalence of a relatively rare condition, which is useful foundational knowledge for individual patient education and population health policy planning. Overall, we found the mean annual incidence and prevalence of any form of uveitis to be 124.3 and 316.4 per 100,000 persons, respectively. Focusing specifically on infectious uveitis, we found the overall mean annual incidence and prevalence of infectious uveitis/scleritis in a U.S.-based population to be 19.1 and 60.6 per 100,000 persons, respectively, or approximately 14% of uveitis/scleritis cases in our sample. In a previous epidemiology study on non-infectious uveitis, Thorne et al [1] estimated that the prevalence of any uveitis was 133 per 100,000, with non-infectious uveitis 121 per 100,000. Thus, the estimated prevalence of infectious uveitis was 12 per 100,000. The present study detected a five-fold greater prevalence of infectious ocular inflammation, which may be due to a variety of reasons. First, as advocated by Borkar et al [14], we employed the use of ancillary Current Procedural Terminology (CPT), National Drug Code (NDC), and Healthcare Common Procedure Coding System (HCPCS) “J” codes for antimicrobial treatment to identify cases of infectious uveitis that may have been coded with an ICD diagnosis code that does not specify an infectious or non-infectious etiology. Second, we included scleritis as a diagnosis of ocular inflammation. Third, the present study employed longitudinal data from 47 million individuals between 2007–2015, whereas the prior study by Thorne et al [1] examined a smaller sample containing only 14 million individuals from a single year, 2012. For these reasons, we believe the present study adds additional information that could not be addressed in the prior analysis.

Nonetheless, our estimates indicate that infectious etiologies represent a smaller share of ocular inflammation as compared with series previously reported in developing countries. This may be for a variety of reasons. The incidence and prevalence of infectious ocular inflammation can vary significantly depending on the causative agent and geography. In the developed world, infectious uveitis can comprise up to 20% of all uveitis cases [5], and our findings confirmed that toxoplasmosis and herpetic infection predominate [11]. In the developing world, infectious uveitis may account for up to 30–50% of all cases of uveitis [11, 15], with the most common etiologies including toxoplasmosis, tuberculosis, onchocerchiasis, and cysticercosis. These estimates of incidence and prevalence fluctuate with clinical setting, emergent infectious patterns, more sensitive diagnostic testing, and novel diseases or disease definitions. Of all the uveitis/scleritis patients included in our U.S.-based study, only 14% were diagnosed with infectious ocular inflammation. The fact that developing countries may have a more rural population with a different set of endemic diseases, in addition to disparities in access to healthcare, likely contributed to the differences between the United States and these developing countries.

Similar to other prior studies of uveitis in the United States [10], we also found the mean age of patients with infectious uveitis/scleritis was greater than the general population, and there was a predilection for female sex. This was further found to be true for all anatomic categories of infectious uveitis/scleritis. Distribution of race, on the other hand, varied significantly depending on the anatomic location of infectious ocular inflammation. African American patients had a higher risk of anterior uveitis (incidence OR = 1.2, p<0.01; prevalence OR = 1.3, p<0.01) and scleritis (incidence OR = 1.2, p = 0.01; prevalence OR = 1.4, p<0.01), whereas Hispanic patients had a higher risk of intermediate uveitis (incidence OR = 2.7, p = 0.05; prevalence OR = 3.2, p = 0.03), and non-Hispanic white patients had a higher risk of posterior uveitis (OR<1 for all other races in comparison to non-Hispanic whites, p<0.01).

The infectious uveitis/scleritis cohort and the general population had a similar distribution of education levels, though it was noted that higher education level conferred a higher relative risk of anterior uveitis and scleritis. We postulate that this could reflect ascertainment bias with enhanced awareness of health and utilization of health care resources in patients who were more educated. More patients in the infectious uveitis/scleritis cohort had less than $100,000 yearly income when compared with the general population; however, there was no significant association between income level and the various types of infectious ocular inflammation after multivariate adjustment. Interestingly, certain geographic regions were associated with different subtypes of infectious ocular inflammation. The New England region had a higher risk of infectious anterior uveitis; the Mountain region had a higher risk of infectious intermediate uveitis; the East South Central and East North Central regions had a higher risk of infectious posterior uveitis; the risk of infectious panuveitis was highest in the South Atlantic and Mountain regions; and the Pacific region had a lower risk of infectious scleritis. Smoking conferred a higher risk for anterior, intermediate and posterior uveitis, but not panuveitis or scleritis. HIV/AIDS was found to be significantly associated with anterior and posterior uveitis. A similar trend was seen in a high HIV prevalence setting in rural South Africa, where 83% of HIV-infected patients had infectious uveitis compared to 51% of HIV-uninfected patients [16]. Rheumatologic disease was associated with an increased risk of anterior uveitis, panuveitis, and scleritis. Many of these patients are on systemic immunomodulatory therapy and may thus be relatively immunocompromised. Of note, diabetes mellitus, which may confer a relative increased susceptibility to endogenous endophthalmitis, was the medical co-morbidity most strongly associated with panuveitis.

Our study was subject to limitations common to the use of data based on medical claims. Chiefly, our study lacked the rich clinical data available in charts. Pimental et al [17] employed chart review to validate identification of uveitis cases by diagnosis codes and found that the positive predictive value of ICD-9 codes was only 61%. To mitigate this limitation, we have taken the measures of requiring two separate visits for infectious uveitis, excluding post-operative cases, and including medication codes as part of our search criteria.

To increase the likelihood of capturing as many valid infectious cases as possible, we leveraged medical and pharmacy claims data to determine infection was present if an anti-microbial medication was used near the index date. In clinical practice, cases of infectious uveitis/scleritis may be treated empirically with antimicrobial medications but ultimately be non-infectious in etiology. As a result, it is likely that some false positive cases may have been included in the study. Furthermore, we may also have missed some cases of infectious uveitis that were not identified until more than a week after initial diagnosis, were not treated with antimicrobials, or did not have specific infectious diagnosis codes. Lastly, it was not possible to create an exact match of one causative infectious organism for each patient, as some patients may have had diagnosis or medication codes mapping to multiple etiologies, and some codes did not identify specific types of infection. Future studies that include chart review may address some of these limitations and be better suited to investigate post-operative ocular infection, an important cause of infectious uveitis that was excluded from the present study.

Also, our sample included only continuously insured individuals with private health care and may not accurately reflect the epidemiology of uveitis in the uninsured or lesser insured populations. Similarly, while the data we used are national and include individuals from all 50 U.S. states, they are not designed to be a representative sample. Despite these limitations, we believe that our analysis provides important new data on the epidemiology of infectious uveitis in the United States. Planned future studies include investigating the incidence and prevalence of specific etiologies of infectious uveitis/scleritis, which we hypothesize would vary significantly among different age groups, geographic locations, and medical co-morbidities, and quantifying the disease burden and health costs associated with ocular inflammation.

Our findings indicate that the incidence and prevalence of infectious ocular inflammation are higher than previously estimated in the United States. Raising awareness about specific demographic and SES factors that confer increased risk for infectious uveitis/scleritis represents an important opportunity to improve diagnosis and treatment of this potentially blinding condition.

## Supporting information

S1 Table Uveitis/scleritis ICD-9 diagnosis codes. (DOCX)

S2 Table Antimicrobial drug names and routes of administration. (DOCX)

S3 Table Definition of census divisions. (DOCX)

S4 Table Multivariable logistic regression and 95% confidence interval of incident cases of infectious ocular inflammation overall and by anatomic category. (DOCX)

S5 Table Multivariable logistic regression and 95% confidence interval of prevalent. cases of infectious ocular inflammation overall and by anatomic category. (DOCX)

S6 Table Mean and median 1- and 5-year incidence of uveitis/scleritis. (DOCX)

S7 Table Prevalence of infectious ocular Inflammation over time. (DOCX)
