# Supplementary material for: Incidence, prevalence, and risk factors of infectious uveitis and scleritis in the United States: A claims-based analysis
Source: PLoS One. 2020 Aug 25;15(8):e0237995. doi: 10.1371/journal.pone.0237995 (PMC7447056; doi:10.1371/journal.pone.0237995)
Supplement: S1 Table — (DOCX) [file pone.0237995.s001.docx]

| **Supplemental table 1**. Uveitis/Scleritis ICD-9 diagnosis codes | |
| --- | --- |
| **Final Diagnosis Codes** | **ICD-9** |
| Anterior uveitis | |
| Acute and subacute iridocyclitis—unspecified | 364.00 |
| Primary iridocyclitis | 364.01 |
| Recurrent iridocyclitis | 364.02 |
| Secondary iridocyclitis, infectious* | 364.03 |
| Secondary iridocyclitis-noninfectious | 364.04 |
| Hypopyon | 364.05 |
| Chronic iridocyclitis, unspecified | 364.1 |
| Chronic iridocyclitis in diseases classified elsewhere | 364.11 |
| Certain types of iridocyclitis | 364.2 |
| Unspecified iridocyclitis uveitis NOS | 364.3 |
| Fuch’s heterochromic cyclitis | 364.21 |
| Herpes zoster iridocyclitis* | 53.22 |
| Herpes simplex iridocyclitis* | 54.44 |
| Glaucomatocyclitic crisis | 364.22 |
| Lens-induced iridocyclitis | 364.23 |
| Syphilis iridocyclitis* | 091.52 |
| Gonococcal iridocyclitis* | 98.41 |
|  |  |
| Intermediate uveitis | |
| Pars planitis/posterior cyclitis | 363.21 |
|  |  |
| Panuveitis/endophthalmitis | |
| Endophthalmitis(global)(infective)(metastatic)(purulent)(subacute)* | 360.0 |
| Unspecified* | 360.00 |
| Acute* | 360.01 |
| Panophthalmitis* | 360.02 |
| Chronic* | 360.03 |
| Vitreous abscess* | 360.04 |
| Parasitic* | 360.13 |
| Phacoanaphylactic | 360.19 |
| Specified type/sympathetic ophthalmia | 360.11 |
| Other endophthalmitis | 360.1 |
| Panuveitis | 360.12 |
| VKH/Harada’s disease (uveomeningeal syndrome) | 363.22, 364.24 |
|  |  |
| Posterior uveitis | |
| Retinitis/chorioretinitis/choroiditis/posterior uveitis—NOS | 363.20 |
| Disseminated/generalized—NOS | 363.10 |
| Posterior pole | 363.11 |
| Peripheral | 363.12 |
| Generalized (with underlying disease) | 363.13 |
| Focal—NOS | 363.00 |
| Juxtapapillary | 363.01 |
| Posterior pole | 363.03 |
| Peripheral | 363.04 |
| Juxtapapillary (neuroretinitis) | 363.05 |
| Paramacular | 363.06 |
| Posterior pole retinitis | 363.07 |
| Peripheral retinitis | 363.08 |
| Retinochoroiditis/retinitis—metastatic | 363.14 |
| Chorioretinitis—due to toxoplasmosis (aquired)* | 130.2 |
| Chorioretinitis—due to toxoplasmosis, congenital (active)* | 771.2 |
| Retinal (peri) vasculitis | 362.18 |
| Retinitis/chorioretinitis—due to histoplasmosis* | 115.92 |
| Capsulatum (American)* | 115.02 |
| Duboisii (African)* | 115.12 |
| Chorioretinitis—syphilitic* | 091.51 |
| Congenital syphilis* | 090.0 |
| Late* | 90.5 |
| Late/central, recurrent* | 95.8 |
| Generalized—in neurosyphilis* | 94.83 |
| Generalized—in secondary syphilis* | 091.51 |
| Chorioretinitis—tuberculosis* | 017.3 |
| Exudative retinitis | 362.12 |
| Disseminated retinitis and retinochoroiditis, pigment epitheliopathy (APMPPE) | 363.15 |
| Behcet’s syndrome | 136.1 |
|  |  |
| Unspecified | |
| Syphilis uveitis* | 091.50 |
|  |  |
| Scleritis |  |
| Episcleritis | 379.01 |
| Scleritis | 379.0 |
| APMPPE = acute posterior multifocal placoid pigment epitheliopathy; ICD = international classification of diseases; NOS = not otherwise specified; VKH = Vogt-Koyanagi-Harada  *infectious etiology | |
