# Supplementary material for: Incidence, prevalence, and risk factors of infectious uveitis and scleritis in the United States: A claims-based analysis
Source: PLoS One. 2020 Aug 25;15(8):e0237995. doi: 10.1371/journal.pone.0237995 (PMC7447056; doi:10.1371/journal.pone.0237995)
Supplement: S2 Table — (DOCX) [file pone.0237995.s002.docx]

| **Supplemental table 2.** Antimicrobial drug names and routes of administration | |
| --- | --- |
| **Antimicrobial** | **Route of Administration** |
| **Systemic** | |
| ACYCLOVIR | PO, IV |
| ALBENDAZOLE | PO |
| AMIKACIN SULFATE | IV |
| AMPHOTERICIN B | PO, IV |
| ATOVAQUONE | PO |
| CEFTAZIDIME | IV |
| CIDOFOVIR | IV |
| CIPROFLOXACIN | PO, IV |
| CLINDAMYCIN | IV |
| DOXYCYCLINE | PO, IV |
| ETHAMBUTOL HCL | PO |
| FAMCICLOVIR | PO |
| FLUCONAZOLE | PO, IV |
| FOMIVIRSEN SODIUM | INTRAOCULAR |
| FOSCARNET SODIUM | IV |
| GANCICLOVIR | INTRAOCULAR, PO, IV |
| GENTAMICIN | IV |
| ISONIAZID | PO |
| IVERMECTIN | PO |
| LEVOFLOXACIN | PO, IV |
| MOXIFLOXACIN | PO, IV |
| OFLOXACIN | PO, IV |
| PENICILLIN G BENZATHINE | IM, IV |
| PYRAZINAMIDE | PO |
| PYRIMETHAMINE | PO |
| PYRIMETHAMINE/SULFADOXINE | PO |
| RIFAMPIN | PO |
| SULFADIAZINE | PO |
| SULFAMETHOXAZOLE/TRIMETHOPRIM | PO, IV |
| THIABENDAZOLE | PO |
| VALACYCLOVIR HCL | PO |
| VALGANCICLOVIR HCL | PO |
| VANCOMYCIN | IV |
| VORICONAZOLE | PO, IV |
| **Intraocular (concurrent CPT 67028)** | |
| AMIKACIN | INJECTION |
| AMPHOTERICIN B | INJECTION |
| CEFTAZIDIME | INJECTION |
| CIDOFOVIR | INJECTION |
| CLINDAMYCIN | INJECTION |
| FOSCARNET SODIUM | INJECTION |
| GENTAMICIN SULFATE | INJECTION |
| VANCOMYCIN HCL | INJECTION |
| VORICONAZOLE | INJECTION |
| CPT = current procedural terminology; IM = intramuscular; IV = intravenous; PO = oral | |
