# Supplementary material for: Incidence, prevalence, and risk factors of infectious uveitis and scleritis in the United States: A claims-based analysis
Source: PLoS One. 2020 Aug 25;15(8):e0237995. doi: 10.1371/journal.pone.0237995 (PMC7447056; doi:10.1371/journal.pone.0237995)
Supplement: S3 Table — (DOCX) [file pone.0237995.s003.docx]

| **Supplemental Table 3.** US Census Divisions | |
| --- | --- |
| **US Census Division** | **States** |
| New England | CT, ME, MA, NH, RI, VT |
| Middle Atlantic | NJ, NY, PA |
| East North Central | IL, IN, MI, OH, WI |
| West North Central | IA, KS, MN, MO, NE, ND, SD |
| South Atlantic | DE, DC, FL, GA, MD, NC, SC, VA, WV |
| East South Central | AL, KY, MS, TN |
| West South Central | AR, LA, OK, TX |
| Mountain | AZ, CO, ID, MT, NV, NM, UT, WY |
| Pacific | AK, CA, HI, OR, WA |
