# Supplementary material for: Incidence, prevalence, and risk factors of infectious uveitis and scleritis in the United States: A claims-based analysis
Source: PLoS One. 2020 Aug 25;15(8):e0237995. doi: 10.1371/journal.pone.0237995 (PMC7447056; doi:10.1371/journal.pone.0237995)
Supplement: S4 Table — (DOCX) [file pone.0237995.s004.docx]

| **Supplemental Table 4.** Multivariable logistic regression and 95% confidence interval of incident cases of infectious ocular inflammation overall and by anatomic category | | | | | | | | | | | | |
| --- | --- | --- | --- | --- | --- | --- | --- | --- | --- | --- | --- | --- |
|  | **Any Uveitis** | | **Scleritis** | | **Anterior Uveitis** | | **Intermediate Uveitis** | | **Posterior Uveitis** | | **Panuveitis** | |
|  | OR | 95% CI | OR | 95% CI | OR | 95% CI | OR | 95% CI | OR | 95% CI | OR | 95% CI |
| ***Age categories*** |  |  |  |  |  |  |  |  |  |  |  |  |
| 0-17 | REF | | REF | | REF | | REF | | REF | | REF | |
| 18-34 | 3.1 | 2.76 3.38 | 5.7 | 3.93 8.23 | 2.9 | 2.47 3.32 | 6.3 | .77 51.25 | 3.7 | 3.07 4.45 | 1.2 | .90 1.64 |
| 35-54 | 6.8 | 6.23 7.52 | 11.6 | 8.20 16.54 | 5.6 | 4.85 6.37 | 8.1 | 1.06 62.65 | 10.2 | 8.61 12.12 | 2.2 | 1.71 2.87 |
| 55-64 | 11.1 | 10.13 12.27 | 14.9 | 10.37 21.31 | 8.3 | 7.21 9.56 | 12.2 | 1.49 100.73 | 18.3 | 15.36 21.71 | 4.8 | 3.74 6.29 |
| 65-74 | 13.7 | 12.34 15.23 | 11 | 7.30 16.52 | 10.1 | 8.64 11.83 | 6 | .38 92.72 | 23.7 | 19.68 28.45 | 6.9 | 5.19 9.23 |
| 75+ | 16.3 | 14.61 18.14 | 10.1 | 6.53 15.50 | 13.4 | 11.40 15.76 | 7.2 | .39 134.36 | 23.3 | 19.26 28.15 | 12.1 | 9.02 16.18 |
| ***Male, n (%)*** | 0.8 | .75 .80 | 0.5 | .47 .58 | 0.8 | .79 .87 | 0.4 | .19 .87 | 0.8 | .72 .79 | 0.9 | .83 1.01 |
| ***Race, n (%)*** |  |  |  |  |  |  |  |  |  |  |  |  |
| Non-Hispanic white | REF | | REF | | REF | | REF | | REF | | REF | |
| Black | 0.9 | .85 .94 | 1.2 | 1.05 1.48 | 1.2 | 1.06 1.25 | 2.3 | .71 7.31 | 0.6 | .56 .67 | 1 | .88 1.23 |
| Hispanic | 0.7 | .66 .75 | 0.8 | .68 1.04 | 0.7 | .67 .81 | 2.7 | 1.02 7.39 | 0.6 | .51 .64 | 1 | .80 1.15 |
| Asian | 0.7 | .61 .74 | 0.8 | .57 1.04 | 0.8 | .68 .91 | 1 | * | 0.5 | .37 .56 | 1 | .73 1.29 |
| Unknown or missing | 1.1 | 1.08 1.19 | 1.2 | 1.07 1.46 | 1.2 | 1.12 1.29 | 2.7 | 1.09 6.74 | 1.1 | .98 1.13 | 1.1 | .95 1.31 |
| ***Education, n (%)*** |  |  |  |  |  |  |  |  |  |  |  |  |
| Less than high school | REF | | REF | | REF | | REF | | REF | | REF | |
| Some college | 1 | .99 1.07 | 1.3 | 1.11 1.46 | 1.1 | 1.04 1.17 | 1.3 | .51 3.31 | 0.9 | .88 .97 | 1.1 | .99 1.26 |
| 4 year college degree or better | 1.1 | 1.08 1.19 | 1.5 | 1.29 1.82 | 1.3 | 1.17 1.37 | 1.3 | .38 4.28 | 0.9 | .87 1.02 | 1.3 | 1.13 1.57 |
| Unknown or missing | 0.8 | .71 .90 | 0.8 | .48 1.36 | 0.8 | .67 1.00 | 1 | * | 0.7 | .60 .89 | 1 | .71 1.41 |
| ***Income, n (%)*** |  |  |  |  |  |  |  |  |  |  |  |  |
| $40K-$49k | REF | | REF | | REF | | REF | | REF | | REF | |
| $50K-$99k | 1.1 | 1.03 1.12 | 1.1 | .97 1.33 | 1.1 | .99 1.13 | 0.5 | .18 1.48 | 1.1 | 1.04 1.17 | 1 | .85 1.10 |
| $100k+ | 1.1 | 1.05 1.16 | 1.3 | 1.12 1.56 | 1.1 | 1.00 1.16 | 1.1 | .40 2.91 | 1.1 | 1.07 1.23 | 0.8 | .72 .98 |
| Unknown or missing | 0.8 | .73 .81 | 0.8 | .69 .99 | 0.7 | .69 .80 | 0.5 | .16 1.49 | 0.8 | .74 .86 | 0.8 | .66 .89 |
| ***Business Product, n (%)*** |  |  |  |  |  |  |  |  |  |  |  |  |
| Medicare | 0.8 | .77 .87 | 0.6 | .49 .79 | 0.8 | .74 .89 | 1 | .13 7.55 | 0.8 | .77 .92 | 0.8 | .68 .97 |
| ***Division, n (%)*** |  |  |  |  |  |  |  |  |  |  |  |  |
| East North Central | REF | | REF | | REF | | REF | | REF | | REF | |
| East South Central | 1.3 | 1.19 1.36 | 1.3 | .98 1.68 | 1.1 | .95 1.25 | 0.8 | .09 7.01 | 1.4 | 1.25 1.48 | 1.1 | .85 1.51 |
| Middle Atlantic | 0.6 | .54 .62 | 1 | .83 1.31 | 1.1 | .97 1.20 | 0.8 | .14 3.94 | 0.3 | .23 .30 | 1 | .81 1.27 |
| Mountain | 0.5 | .49 .56 | 0.9 | .72 1.13 | 0.9 | .81 1.00 | 2.1 | .68 6.69 | 0.3 | .22 .28 | 1.3 | 1.07 1.60 |
| New England | 0.6 | .54 .64 | 1 | .71 1.28 | 1.2 | 1.06 1.36 | 1 | * | 0.2 | .19 .26 | 1.2 | .96 1.58 |
| Pacific | 0.4 | .37 .43 | 0.7 | .56 .90 | 0.8 | .75 .92 | 0.9 | .23 3.66 | 0.1 | .13 .17 | 0.8 | .62 .96 |
| South Atlantic | 0.8 | .74 .80 | 1.1 | .96 1.33 | 1.2 | 1.13 1.32 | 0.7 | .21 2.19 | 0.5 | .44 .50 | 1.5 | 1.28 1.77 |
| West North Central | 0.2 | .91 1.01 | 1.2 | .94 1.41 | 1.2 | 1.05 1.27 | 1 | .22 4.08 | 0.9 | .79 .91 | 1 | .85 1.30 |
| West South Central | 1 | .66 .74 | 1 | .85 1.24 | 1.2 | 1.09 1.31 | 0.5 | .11 2.07 | 0.4 | .38 .45 | 1 | .84 1.29 |
| ***Smoking, n (%)*** | 1.2 | 1.06 1.26 | 1 | .71 1.37 | 1.3 | 1.14 1.48 | 1.6 | .21 12.45 | 1.1 | .99 1.29 | 0.9 | .64 1.18 |
| ***Comorbidities*** |  |  |  |  |  |  |  |  |  |  |  |  |
| Congestive Heart Failure | 0.9 | .79 .96 | 1 | .71 1.52 | 1 | .85 1.13 | 1 | * | 0.8 | .65 .89 | 0.8 | .62 1.03 |
| Cardiac Arrhythmia | 1.1 | 1.06 1.21 | 1.2 | .88 1.51 | 1.2 | 1.10 1.35 | 1 | * | 1 | .93 1.15 | 1.1 | .96 1.37 |
| Valvular Disease | 1.1 | 1.05 1.26 | 1.5 | 1.11 2.10 | 0.9 | .81 1.09 | 3.4 | .43 26.62 | 1.3 | 1.11 1.46 | 1.3 | 1.01 1.63 |
| Peripheral Vascular Disorders | 1 | .88 1.04 | 0.9 | .59 1.29 | 0.9 | .83 1.08 | 2.2 | .26 18.95 | 0.9 | .83 1.09 | 1 | .84 1.29 |
| Hypertension | 1.3 | 1.21 1.31 | 1.4 | 1.24 1.66 | 1.4 | 1.27 1.44 | 0.9 | .28 2.93 | 1.2 | 1.10 1.24 | 1.2 | 1.08 1.38 |
| Other Neurological Disorders | 1.3 | 1.13 1.39 | 1.2 | .82 1.85 | 1.3 | 1.07 1.50 | 3.1 | .40 24.34 | 1.2 | .99 1.39 | 1.5 | 1.11 1.92 |
| Chronic Pulmonary Disease | 1.3 | 1.22 1.36 | 1.5 | 1.19 1.78 | 1.4 | 1.28 1.53 | 1 | * | 1.3 | 1.16 1.38 | 0.9 | .79 1.13 |
| Diabetes | 1.3 | 1.28 1.40 | 1.2 | .98 1.40 | 1.2 | 1.10 1.28 | 0.7 | .15 3.60 | 1.3 | 1.22 1.41 | 2.3 | 2.01 2.57 |
| Hypothyroidism | 1.1 | 1.04 1.17 | 1.4 | 1.14 1.66 | 1.1 | 1.00 1.20 | 0.5 | .07 3.86 | 1.1 | .97 1.16 | 1.1 | .94 1.32 |
| Renal Failure | 1.1 | 1.03 1.22 | 1 | .67 1.42 | 1.1 | 1.01 1.31 | 5.7 | 1.04 31.15 | 1.1 | .93 1.22 | 1.2 | .99 1.48 |
| Liver Disease | 1.2 | 1.08 1.37 | 1.5 | .99 2.19 | 1.3 | 1.04 1.52 | 6.2 | 1.34 28.93 | 1 | .84 1.27 | 1.5 | 1.09 2.07 |
| Peptic Ulcer Disease | 1.1 | .86 1.47 | 2.1 | 1.00 4.49 | 0.9 | .58 1.48 | 1 | * | 1.2 | .77 1.80 | 1.1 | .51 2.29 |
| AIDS/HIV | 5.6 | 4.58 6.73 | 2.7 | 1.14 6.63 | 4.5 | 3.31 6.24 | 1 | * | 8.9 | 6.86 11.43 | 1.1 | .26 4.24 |
| Cancer | 1.3 | 1.19 1.37 | 1.1 | .85 1.52 | 1.2 | 1.11 1.39 | 4.3 | 1.21 15.43 | 1.4 | 1.23 1.52 | 1.1 | .93 1.42 |
| Rheumatologic Disease | 1.8 | 1.67 1.96 | 2.8 | 2.23 3.59 | 2.1 | 1.88 2.39 | 1.9 | .26 14.55 | 1.4 | 1.24 1.63 | 1.6 | 1.23 2.07 |
| Obesity | 1.1 | 1.03 1.22 | 1.4 | 1.04 1.78 | 1.1 | .92 1.22 | 1.4 | .17 10.51 | 1.1 | 1.01 1.31 | 1 | .79 1.36 |
| Weight Loss | 1.4 | 1.20 1.56 | 1.3 | .73 2.16 | 1.3 | 1.02 1.57 | 4.6 | .55 38.50 | 1.3 | 1.04 1.61 | 1.9 | 1.37 2.56 |
| Fluid and Electrolyte Disorders | 1.3 | 1.16 1.39 | 1.1 | .80 1.63 | 1.4 | 1.20 1.57 | 3.9 | .75 20.23 | 1.1 | .94 1.27 | 1.5 | 1.23 1.95 |
| Anemia | 1.1 | 1.04 1.26 | 1.2 | .87 1.75 | 1.2 | 1.03 1.39 | 1 | * | 1 | .89 1.23 | 1.2 | .93 1.54 |
| Substance Abuse | 1 | .81 1.14 | 0.5 | .25 1.14 | 1 | .76 1.29 | 1 | * | 0.9 | .65 1.16 | 1.6 | 1.08 2.53 |
| Psychoses | 1 | .86 1.24 | 1.1 | .52 2.14 | 0.8 | .61 1.16 | 1 | * | 1.4 | 1.06 1.79 | 0.7 | .39 1.25 |
| Depression | 1.1 | 1.05 1.19 | 1.6 | 1.33 1.93 | 1.2 | 1.07 1.30 | 1.2 | .28 5.17 | 1 | .89 1.09 | 1.1 | .86 1.30 |
| Other | 1.2 | 1.04 1.28 | 1 | .66 1.55 | 1.1 | .93 1.30 | 1 | * | 1.1 | .92 1.30 | 1.6 | 1.26 2.11 |
| OR = odds ratio; CI = confidence interval; REF = reference; AIDS = acquired immunodeficiency syndrome; HIV = human immunodeficiency virus; * = omitted from the regression due to small sample size | | | | | | | | | | | | |
| Data for any infectious uveitis/scleritis is shaded in purple. Data for each category of infectious uveitis/scleritis is shaded in green. | | | | | | | | | | | | |
