# Supplementary material for: Incidence, prevalence, and risk factors of infectious uveitis and scleritis in the United States: A claims-based analysis
Source: PLoS One. 2020 Aug 25;15(8):e0237995. doi: 10.1371/journal.pone.0237995 (PMC7447056; doi:10.1371/journal.pone.0237995)
Supplement: S5 Table — (DOCX) [file pone.0237995.s005.docx]

| **Supplemental Table 5.** Multivariable logistic regression and 95% confidence interval of prevalent cases of infectious ocular inflammation overall and by anatomic category | | | | | | | | | | | | |
| --- | --- | --- | --- | --- | --- | --- | --- | --- | --- | --- | --- | --- |
|  | **Any Uveitis** | | **Scleritis** | | **Anterior Uveitis** | | **Intermediate Uveitis** | | **Posterior Uveitis** | | **Panuveitis** | |
|  | OR | 95% CI | OR | 95% CI | OR | 95% CI | OR | 95% CI | OR | 95% CI | OR | 95% CI |
| ***Age categories*** |  |  |  |  |  |  |  |  |  |  |  |  |
| 0-17 | REF | | REF | | REF | | REF | | REF | | REF | |
| 18-34 | 3.3 | 2.85 3.80 | 5.4 | 3.15 9.12 | 2.9 | 2.37 3.51 | 6.6 | .81 54.18 | 3.7 | 2.86 4.74 | 2.5 | 1.41 4.43 |
| 35-54 | 8 | 6.98 9.08 | 11.9 | 7.17 19.64 | 6.5 | 5.45 7.79 | 5.1 | .64 41.35 | 10.3 | 8.15 12.97 | 4.9 | 2.90 8.22 |
| 55-64 | 12.7 | 11.15 14.57 | 15.8 | 9.43 26.45 | 9.9 | 8.22 11.88 | 12.5 | 1.51 103.05 | 18.2 | 14.35 22.96 | 7.9 | 4.65 13.46 |
| 65-74 | 15.6 | 13.47 18.01 | 12.2 | 6.86 21.72 | 11.4 | 9.32 13.98 | 5.8 | .35 93.66 | 23.6 | 18.41 30.38 | 15.2 | 8.74 26.39 |
| 75+ | 18.4 | 15.89 21.42 | 9.9 | 5.38 18.33 | 15.2 | 12.39 18.77 | 7.4 | .38 145.50 | 20.6 | 15.89 26.73 | 31.7 | 18.22 55.24 |
| ***Male, n (%)*** | 0.8 | .74 .80 | 0.5 | .45 .61 | 0.8 | .79 .88 | 0.5 | .22 1.10 | 0.7 | .69 .79 | 0.9 | .81 1.07 |
| ***Race, n (%)*** |  |  |  |  |  |  |  |  |  |  |  |  |
| Non-Hispanic white | REF | | REF | | REF | | REF | | REF | | REF | |
| Black | 1 | .91 1.04 | 1.4 | 1.08 1.72 | 1.3 | 1.15 1.39 | 1.9 | .50 6.95 | 0.6 | .54 .69 | 1.2 | .92 1.44 |
| Hispanic | 0.7 | .65 .77 | 0.9 | .71 1.25 | 0.7 | .64 .82 | 3.2 | 1.15 8.75 | 0.6 | .50 .68 | 0.9 | .72 1.22 |
| Asian | 0.7 | .60 .78 | 0.7 | .43 1.11 | 0.8 | .67 .96 | 1 | * | 0.5 | .37 .64 | 0.9 | .57 1.35 |
| Unknown or missing | 1.2 | 1.09 1.23 | 1.3 | 1.01 1.58 | 1.2 | 1.11 1.33 | 2.5 | .87 6.98 | 1.1 | 1.00 1.21 | 1 | .79 1.27 |
| ***Education, n (%)*** |  |  |  |  |  |  |  |  |  |  |  |  |
| Less than high school | REF | | REF | | REF | | REF | | REF | | REF | |
| Some college | 1.1 | 1.02 1.12 | 1.2 | .96 1.38 | 1.1 | 1.07 1.23 | 1 | .39 2.67 | 1 | .90 1.04 | 1.1 | .96 1.34 |
| 4 year college degree or better | 1.1 | 1.07 1.22 | 1.3 | 1.04 1.69 | 1.3 | 1.19 1.45 | 0.6 | .14 2.29 | 1 | .86 1.06 | 1.2 | .91 1.48 |
| Unknown or missing | 0.8 | .65 .90 | 0.9 | .44 1.70 | 0.8 | .59 .98 | 1 | * | 0.7 | .50 .88 | 1.2 | .73 1.89 |
| ***Income, n (%)*** |  |  |  |  |  |  |  |  |  |  |  |  |
| $40K-$49k | REF | | REF | | REF | | REF | | REF | | REF | |
| $50K-$99k | 1.1 | 1.02 1.13 | 1.1 | .87 1.32 | 1.1 | .99 1.16 | 0.7 | .23 2.18 | 1.1 | 1.00 1.18 | 1 | .86 1.22 |
| $100k+ | 1.1 | 1.03 1.16 | 1.2 | .94 1.48 | 1.1 | .99 1.19 | 1.7 | .57 4.99 | 1.1 | 1.01 1.22 | 0.9 | .71 1.11 |
| Unknown or missing | 0.7 | .70 .80 | 0.8 | .60 .98 | 0.7 | .65 .79 | 0.4 | .11 1.70 | 0.8 | .70 .87 | 0.8 | .63 .97 |
| ***Business Product, n (%)*** |  |  |  |  |  |  |  |  |  |  |  |  |
| Medicare | 0.8 | .74 .87 | 0.6 | .42 .81 | 0.8 | .73 .92 | 1.5 | .16 13.79 | 0.8 | .68 .88 | 0.9 | .70 1.16 |
| ***Division, n (%)*** |  |  |  |  |  |  |  |  |  |  |  |  |
| East North Central | REF | | REF | | REF | | REF | | REF | | REF | |
| East South Central | 1.2 | 1.06 1.26 | 1.2 | .86 1.81 | 0.9 | .80 1.12 | 1 | .12 9.46 | 1.3 | 1.12 1.41 | 1.3 | .90 2.01 |
| Middle Atlantic | 0.6 | .53 .63 | 0.8 | .58 1.14 | 1 | .91 1.18 | 1 | .18 5.45 | 0.3 | .22 .31 | 1 | .72 1.41 |
| Mountain | 0.5 | .48 .57 | 0.7 | .52 1.01 | 0.9 | .79 1.02 | 2.1 | .57 7.59 | 0.2 | .20 .27 | 1.4 | 1.04 1.87 |
| New England | 0.7 | .59 .73 | 0.9 | .58 1.35 | 1.3 | 1.10 1.47 | 1 | * | 0.2 | .17 .27 | 1.5 | 1.07 2.11 |
| Pacific | 0.4 | .38 .45 | 0.6 | .44 .87 | 0.8 | .73 .94 | 1.2 | .28 5.44 | 0.1 | .11 .15 | 0.9 | .66 1.24 |
| South Atlantic | 0.8 | .75 .84 | 1.1 | .89 1.40 | 1.2 | 1.09 1.32 | 0.9 | .26 3.21 | 0.5 | .43 .52 | 1.6 | 1.22 1.97 |
| West North Central | 0.2 | .93 1.06 | 1.3 | .96 1.66 | 1.1 | 1.01 1.27 | 0.9 | .16 4.84 | 0.9 | .78 .94 | 1.4 | 1.06 1.88 |
| West South Central | 1 | .65 .75 | 1.1 | .83 1.40 | 1.1 | .99 1.24 | 0.4 | .07 2.25 | 0.4 | .36 .45 | 1.1 | .81 1.49 |
| ***Smoking, n (%)*** | 1.1 | 1.01 1.27 | 0.9 | .58 1.48 | 1.2 | 1.06 1.47 | 2.2 | .28 17.27 | 1.1 | .92 1.32 | 0.9 | .58 1.36 |
| ***Comorbidities - 12 months pre-index*** |  |  |  |  |  |  |  |  |  |  |  |  |
| Congestive Heart Failure | 0.9 | .78 1.00 | 0.6 | .29 1.10 | 1 | .83 1.17 | 1 | * | 0.8 | .61 .95 | 0.9 | .64 1.25 |
| Cardiac Arrhythmia | 1.1 | 1.05 1.25 | 1.1 | .76 1.65 | 1.3 | 1.13 1.44 | 1 | * | 1.1 | .91 1.22 | 1 | .78 1.29 |
| Valvular Disease | 1.1 | .98 1.25 | 1.4 | .89 2.30 | 0.9 | .77 1.10 | 4.1 | .51 33.20 | 1.3 | 1.08 1.58 | 1.2 | .86 1.68 |
| Peripheral Vascular Disorders | 0.9 | .83 1.04 | 1 | .58 1.67 | 0.9 | .79 1.10 | 2.7 | .30 23.61 | 0.9 | .74 1.10 | 1 | .72 1.31 |
| Hypertension | 1.3 | 1.24 1.37 | 1.3 | 1.10 1.65 | 1.4 | 1.28 1.49 | 0.9 | .24 3.08 | 1.3 | 1.15 1.36 | 1.2 | 1.00 1.40 |
| Other Neurological Disorders | 1.1 | .98 1.31 | 1.6 | .98 2.73 | 1.1 | .92 1.39 | 4.1 | .53 32.33 | 1 | .76 1.27 | 1.3 | .86 1.87 |
| Chronic Pulmonary Disease | 1.3 | 1.20 1.39 | 1.4 | 1.07 1.90 | 1.5 | 1.31 1.61 | 1 | * | 1.2 | 1.06 1.35 | 0.9 | .70 1.16 |
| Diabetes | 1.3 | 1.19 1.34 | 1.1 | .84 1.41 | 1.1 | 1.05 1.25 | 1 | .21 5.17 | 1.3 | 1.16 1.41 | 2 | 1.64 2.32 |
| Hypothyroidism | 1.1 | 1.03 1.20 | 1.4 | 1.07 1.82 | 1.1 | .99 1.23 | 0.7 | .09 5.07 | 1.1 | .98 1.25 | 1 | .78 1.27 |
| Renal Failure | 1.1 | 1.00 1.25 | 1 | .60 1.73 | 1.1 | .98 1.34 | 2.8 | .30 25.88 | 1 | .84 1.24 | 1.2 | .94 1.63 |
| Liver Disease | 1.3 | 1.13 1.53 | 1.4 | .81 2.51 | 1.2 | .99 1.57 | 3.5 | .43 28.17 | 1.3 | 1.04 1.72 | 1.5 | .96 2.37 |
| Peptic Ulcer Disease | 1.2 | .85 1.68 | 1.8 | .56 5.52 | 0.9 | .50 1.57 | 1 | * | 1.5 | .86 2.48 | 1.4 | .58 3.42 |
| AIDS/HIV | 6.4 | 5.09 8.09 | 3.2 | 1.03 10.05 | 4.8 | 3.27 6.92 | 1 | * | 11.2 | 8.25 15.30 | 1.1 | .16 8.13 |
| Cancer | 1.3 | 1.22 1.46 | 1 | .66 1.56 | 1.3 | 1.14 1.49 | 5.6 | 1.54 20.39 | 1.4 | 1.22 1.62 | 1.3 | .99 1.71 |
| Rheumatoid Arthritis/collagen | 1.9 | 1.69 2.08 | 2.9 | 2.08 4.01 | 2.2 | 1.94 2.58 | 2.5 | .33 19.34 | 1.3 | 1.11 1.64 | 1.7 | 1.18 2.40 |
| Obesity | 1.1 | .94 1.18 | 1.5 | 1.03 2.16 | 1 | .82 1.16 | 1 | * | 1.1 | .89 1.29 | 1 | .66 1.49 |
| Weight Loss | 1.4 | 1.19 1.67 | 1.5 | .71 2.99 | 1.2 | .94 1.59 | 6.9 | .82 58.33 | 1.4 | 1.05 1.90 | 1.9 | 1.28 2.91 |
| Fluid and Electrolyte Disorders | 1.4 | 1.24 1.56 | 1.1 | .68 1.87 | 1.5 | 1.27 1.75 | 2.3 | .25 20.20 | 1.2 | .95 1.43 | 1.9 | 1.40 2.52 |
| Anemia | 1.1 | .98 1.26 | 1.4 | .85 2.19 | 1.2 | .99 1.42 | 1 | * | 0.9 | .74 1.18 | 1.2 | .87 1.73 |
| Substance Abuse | 0.9 | .72 1.15 | 1 | .43 2.25 | 1 | .70 1.33 | 1 | * | 0.6 | .40 1.00 | 1.8 | 1.01 3.17 |
| Psychoses | 1 | .76 1.26 | 0.7 | .24 2.37 | 0.8 | .55 1.21 | 1 | * | 1.3 | .92 1.96 | 0.8 | .42 1.73 |
| Depression | 1.1 | 1.01 1.19 | 1.4 | 1.05 1.81 | 1.2 | 1.03 1.31 | 0.8 | .10 5.71 | 0.9 | .81 1.07 | 1.3 | .96 1.66 |
| Other | 1.1 | .99 1.31 | 1.2 | .66 2.11 | 1.1 | .86 1.29 | 1 | * | 1.1 | .89 1.44 | 1.6 | 1.10 2.24 |
| OR = odds ratio; CI = confidence interval; AIDS = acquired immunodeficiency syndrome; HIV = human immunodeficiency virus; * = omitted from the regression due to small sample size | | | | | | | | | | | | |
| Data for any infectious uveitis/scleritis is shaded in purple. Data for each category of infectious uveitis/scleritis is shaded in green. | | | | | | | | | | | | |
