# Supplementary material for: Incidence, prevalence, and risk factors of infectious uveitis and scleritis in the United States: A claims-based analysis
Source: PLoS One. 2020 Aug 25;15(8):e0237995. doi: 10.1371/journal.pone.0237995 (PMC7447056; doi:10.1371/journal.pone.0237995)
Supplement: S6 Table — (DOCX) [file pone.0237995.s006.docx]

| **Supplemental Table 6**. Mean and median 1- and 5-year incidence of uveitis/scleritis | | | | |
| --- | --- | --- | --- | --- |
|  | 1-year incidence (per 100,000) | | 5-year incidence (per 100,000) | |
|  | Mean | Median | Mean | Median |
| Any ocular inflammation | 18.9 | 19.2 | 85.4 | 86.9 |
| Scleritis | 1.6 | 1.6 | 7.6 | 7.6 |
| Any uveitis | 21.6 | 21.8 | 77.8 | 78.7 |
| Anterior uveitis | 7.5 | 7.5 | 34.8 | 35.7 |
| Intermediate uveitis | 0.03 | 0.03 | 0.2 | 0.2 |
| Posterior uveitis | 8.0 | 7.8 | 36.2 | 36.2 |
| Panuveitis | 1.8 | 1.8 | 8.1 | 8.1 |
