# Supplementary material for: Incidence, prevalence, and risk factors of infectious uveitis and scleritis in the United States: A claims-based analysis
Source: PLoS One. 2020 Aug 25;15(8):e0237995. doi: 10.1371/journal.pone.0237995 (PMC7447056; doi:10.1371/journal.pone.0237995)
Supplement: S7 Table — (DOCX) [file pone.0237995.s007.docx]

| **Supplemental Table 7**. Prevalence of Infectious Ocular Inflammation Over Time | | | | | | | | | |
| --- | --- | --- | --- | --- | --- | --- | --- | --- | --- |
| Year | 2007 | 2008 | 2009 | 2010 | 2011 | 2012 | 2013 | 2014 | 2015 |
| Any ocular inflammation | 8.4 | 18.3 | 27.8 | 38.9 | 49.9 | 61.1 | 71.8 | 91.7 | 125.0 |
| Scleritis | 0.7 | 1.5 | 2.3 | 3.2 | 4.0 | 4.9 | 5.5 | 6.8 | 9.0 |
| Any uveitis | 7.8 | 20.4 | 33.2 | 46.5 | 60.2 | 74.5 | 88.2 | 102.1 | 113.6 |
| Anterior uveitis | 4.5 | 8.7 | 13.2 | 18.3 | 23.1 | 28.0 | 32.7 | 41.6 | 56.3 |
| Intermediate uveitis | 0.0 | 0.0 | 0.1 | 0.1 | 0.1 | 0.2 | 0.2 | 0.3 | 0.3 |
| Posterior uveitis | 2.7 | 6.7 | 10.3 | 14.6 | 19.1 | 23.5 | 27.9 | 35.7 | 49.3 |
| Panuveitis | 0.5 | 1.2 | 1.9 | 2.6 | 3.4 | 4.4 | 5.2 | 7.0 | 9.8 |
